# How do the general population behave with facemasks to prevent COVID-19 in the community? A multi-site observational study

**DOI:** 10.1186/s13756-021-00927-6

**Published:** 2021-03-29

**Authors:** Colin Deschanvres, Thomas Haudebourg, Nathan Peiffer-Smadja, Karine Blanckaert, David Boutoille, Jean-Christophe Lucet, Gabriel Birgand

**Affiliations:** 1 Centre D’Appui À La Prévention Des Infections Associées Aux Soins (CPias) Des Pays de La Loire, Nantes University Hospital, 5 rue du Professeur Yves Boquien, 44000 Nantes, France; 2 Infectious Disease Department, Nantes University Hospital, 44000 Nantes, France; 3 INSERM, IAME, UMR 1137, Univ Paris Diderot, Sorbonne Paris Cité, 75018 Paris, France; 4 AP-HP, Hôpital Bichat – Claude Bernard, Infection Control Unit, 75018 Paris, France; 5 NIHR Health Protection Research Unit in Healthcare Associated Infection and Antimicrobial Resistance At Imperial College London, Hammersmith Campus, Du Cane Road, London, UK

**Keywords:** Facemask behaviour, Clothmask, COVID-19, Infection control, Community

## Abstract

**Objective:**

The appropriate use of facemasks, recommended or mandated by authorities, is critical to prevent the spread of COVID-19 in the community. We aim to evaluate frequency and quality of facemask use in general populations.

**Methods:**

A multi-site observational study was carried out from June to July 2020 in the west of France. An observer was positioned at a predetermined place, facing a landmark, and all individual passing between the observer and the landmark were included. The observer collected information on facemask use (type, quality of positioning), location and demographic characteristics.

**Results:**

A total of 3354 observations were recorded. A facemask was worn by 56.4% (n = 1892) of individuals, including surgical facemasks (56.8%, n = 1075) and cloth masks (43.2%, n = 817). The facemask was correctly positioned in 75.2% (n = 1422) of cases. The factors independently associated with wearing a facemask were being indoors (adjusted odds ratio [aOR], 2.7; 95% confidence interval [CI] 2.28–3.19), being in a mandatory area (aOR, 6.92; 95% CI 5–9.7), female gender (aOR, 1.75; 95% CI 1.54–2.04), age 41–65 years (aOR, 1.7; 95% CI 1.43–2.02) and age > 65 years (aOR, 2.28; 95% CI 1.83–2.85). The factors independently associated with correct mask position were rural location (aOR, 1.38; 95% CI 1.07–1.79), being in an indoor area (aOR, 1.85; 95% CI 1.49–2.3), use of clothmask (aOR, 1.53; 95% CI 1.23–1.91), and age > 40 years (aOR, 1.75 95%CI 1.37–2.23).

**Conclusions:**

During the initial phase of the COVID-19 pandemic, the frequency and quality of facemask wearing remained low in the community setting. Young people in general, and men in particular, represent the priority targets for information campaigns. Simplifying the rules to require universal mandatory facemasking seemed to be the best approach for health authorities.

**Supplementary Information:**

The online version contains supplementary material available at 10.1186/s13756-021-00927-6.

## Background

Since the emergence of the Coronavirus (COVID-19) epidemic, wearing a facemask in the community has become commonplace. In many countries, facemasks are mandatory in crowded areas where social distancing cannot be respected and are recommended outdoors [1].Appropriate use of facemasks is critical for protection in the community to prevent the spread of COVID-19 [2]. However, the constraints and discomfort caused in a population unfamiliar with this protective equipment can result in suboptimal use, leading to ineffective protection against COVID-19. Observation and quantification of the quality of facemask use is required to: assess the level of respiratory protection, inform decision makers on the effectiveness of measures, and identify levers for behavior change. We evaluated the frequency and the quality of facemask use in the general populations with different socio-spatial backgrounds, and contextual factors associated with the appropriate use of the facemask.

## Methods

From June 25, 2020, to July 21, 2020, we conducted observations in 13 cities and 43 different locations in the Pays de la Loire region in western France with a population of 3.8 million (Additional file 1: Fig. S1). The observations were performed in various areas: rural and urban (cities with > 10,000 and with < 10,000 inhabitants), indoors (shopping centers, train stations) or outdoors (shopping streets), and in areas where facemasks were or were not mandatory. The observer was positioned in a predetermined place, facing a landmark, and all people passing between the observer and the landmark were included. For each individual, the researcher recorded if a facemask was worn, the type of facemask, and the quality of facemask positioning.

The primary outcome of this study was the correct positioning of the facemask. Secondary outcomes were the frequency of mask wearing and factors associated with the frequency and correct positioning of facemask wearing. The face mask was considered to be worn if it was placed on the face, regardless of its positioning. The facemask was considered incorrectly worn if it was in one of the following positions: below the nose, below the mouth, on the forehead, on one ear, on backward (outside in), with no adjustment of the bar on the nose, not stretched under the chin, cross fasteners (twisted elastic, strap from top to bottom), partial attachment with only one strap on each side or with long hair falling on the mask. (Additional file 2: Fig. S2) For each observation session, information on the time, location, and mandatory status was recorded. In addition, the gender was collected and the age category was estimated (21–40, 41–65, and > 65 years). The data were collected on a smartphone using a Google form. Contingency tables and chi-squared tests were used for categorical variables. Unadjusted Odds Ratio (ORs) were determined and 95% confidence intervals (95% CI) were computed. Multiple logistic regression was performed. Variables associated with *p* values < 0.25 in the bivariate analysis were entered into the model to obtain maximum likelihood estimates. These analyses were performed using R version 3.6.1.

### Results

A total of 3354 observations were performed during 55 sessions (Table 1): 1639 (49%) observations were performed indoors and 1715 (51%) outdoors. The ratio of males to females was 0.73, and 44.6% (*n* = 1495) were aged 21–40 years, 35.3% (*n* = 1184) were aged 41–65 years, and 20.1% (*n* = 675) were > 65 years.

**Table 1:** Description of the study population, with demographic characteristics, frequency and qualitative characteristics of use of masks

Characteristics	Overall, n (%)	Outdoor, n (%)		Indoor, n (%)	
		Urban	Rural	Urban	Rural
Number of observations	3354	1165	550	974	665
Gender					
Female	1943 (57.9)	705 (60.5)	303 (55.1)	550 (56.5)	385 (57.9)
Male	1411 (42.1)	460 (39.5)	247 (44.9)	424 (43.5)	280 (42.1)
Age category					
21–40 years	1495 (44.6)	705 (60.5)	141 (25.6)	456 (46.8)	193 (29)
41–65 years	1184 (35.3)	373 (32)	190 (34.5)	365 (37.5)	256 (38.5)
> 65 years	675 (20.1)	87 (7.5)	219 (39.8)	153 (15.7)	216 (32.5)
Time of day					
Morning	1454 (43.4)	269 (23.1)	400 (72.7)	328 (33.7)	457 (68.7)
Afternoon	1900 (56.6)	896 (76.9)	150 (27.3)	646 (66.3)	208 (31.3)
Mask mandated					
No	2773 (82.7)	1165 (100)	550 (100)	510 (52.4)	548 (82.4)
Yes	581 (17.3)	0 (0)	0 (0)	464 (47.6)	117 (17.6)
Presence of a facemask					
No	1462 (43.6)	732 (62.8)	304 (55.3)	235 (24.1)	191 (28.7)
Yes	1892 (56.4)	433 (37.2)	246 (44.7)	739 (75.9)	474 (71.3)
Type of facemask (n = 1892)					
Surgical facemask	1075 (56.8)	266 (61.4)	131 (53.3)	419 (56.7)	259 (54.6)
Cloth mask	817 (43.2)	167 (38.6)	115 (46.7)	320 (43.3)	215 (45.4)
Quality of mask positioning (n = 1892)					
Correct	1422 (75.2)	264 (61)	191 (77.6)	576 (77.9)	391 (82.5)
Incorrect	470 (24.8)	169 (39)	55 (22.4)	163 (22.1)	83 (17.5)
Incorrect positioning (n = 470)					
Below the mouth	141 (30)	82 (48.5)	14 (25.5)	40 (24.5)	5 (6)
Below the nose	130 (27.7)	37 (21.9)	23 (41.8)	37 (22.7)	33 (39.8)
Cross straps	61 (13)	8 (4.7)	6 (10.9)	31 (19)	16 (19.3)
Not adjusted on the nose	43 (9.1)	10 (5.9)	3 (5.5)	24 (14.7)	6 (7.2)
Hair down on face	33 (7)	17 (10.1)	5 (9.1)	5 (3.1)	6 (7.2)
Partial mask attachment with strap	35 (7.4)	9 (5.3)	1 (1.8)	13 (8)	12 (14.5)
Not stretched under the chin	13 (2.8)	2 (1.2)	1 (1.8)	8 (4.9)	2 (2.4)
On one ear	10 (2.1)	1 (0.6)	2 (3.6)	4 (2.5)	3 (3.6)
On the forehead	3 (0.6)	3 (1.8)	0 (0)	0 (0)	0 (0)
Worn backward	1 (0.2)	0 (0)	0 (0)	1 (0.6)	0 (0)

A facemask was worn by 56.4% (*n* = 1892) of individuals, varying from 40% (*n* = 679) outdoors and 74% (*n* = 1213) indoors, 59% (*n* = 720) in rural areas, 55% (*n* = 1172) in urban areas, 49% (*n* = 1359) in non-mandatory areas, and 92% (*n* = 533) in mandatory areas. With regard to the type of facemask worn, 56.8% (*n* = 1075) wore a surgical facemask and 43.2% (*n* = 817) wore a cloth mask. For the main outcome, among the 1892 individuals wearing a facemask, 75.2% (*n* = 1422) were wearing it correctly. Overall, 42.4% (n = 1422 of 3354) of the population studied was effectively protected by the correct use of the facemask. Of the 470 facemasks positioned incorrectly, 141 (30%) were below the mouth and 130 (27.7%) below the nose.

In the multivariate analysis, facemasks were significantly more often worn indoors (adjusted odds ratio [aOR], 2.7 (2.28–3.19); 95% CI 0.31–0.44; *p* < 0.001), in mandatory areas (aOR, 6.92; 95% CI 5–9.7; *p* < 0.001) and by older individuals aged > 65 years (aOR, 2.28; 95% CI 1.83–2.85; *p* < 0.001) and those aged 41–65 years (aOR, 1.7; 95% CI 1.43–2.02; *p* = 0.008). Facemasks were significantly less frequently worn by males (aOR, 0.57; 95% CI 0.49–0.75; *p* < 0.001) (Table 2).

**Table 2:** Univariate and multivariable analysis of factors influencing the use and the visual correct position of facemask fit

Factors	Facemask	No facemask	Univariate OR (95% CI)	*p*	Multivariate aOR (95% CI)	*p*	Correct position	Incorrect position	Univariate OR (95% CI)	*p*	Multivariate aOR (95% CI)	*p*
Number	1892 (56.4)	1462 (43.6)					1422 (75.2)	470 (24.8)				
Area												
Urban	1172 (54.8)	967 (45.2)	Reference		Reference		840 (71.7)	332 (28.3)	Reference		Reference	
Rural	720 (59.3)	495 (40.7)	1.2 (1.04–1.39)	0.012	1.18 (0.98–1.41)	0.075	582 (80.8)	138 (19.2)	1.67 (1.33–2.08)	< 0.001	1.38 (1.07–1.79)	0.03
Location												
Outdoor	679 (39.6)	1036 (60.4)	Reference		Reference		455 (67)	224 (33)	Reference		Reference	
Indoor	1213 (74)	426 (26)	4.35 (3.7–5)	< 0.001	2.7 (2.28–3.19)	< 0.001	967 (79.7)	246 (20.3)	1.92 (1.56–2.38)	< 0.001	1.85 (1.49–2.3)	< 0.001
Mandatory												
No	1359 (49)	1414 (51)	Reference		Reference		1016 (74.8)	343 (25.2)	Reference			
Yes	533 (91.7)	48 (8.3)	11.11 (8.33–16.67)	< 0.001	6,92 (5–9,7)	< 0.001	406 (76.2)	127 (23.8)	1.08 (0.85–1.37)	0.52		
Time of day												
Morning	800 (55)	654 (45)	Reference		Reference		641 (80.1)	159 (19.9)	Reference		Reference	
Afternoon	1092 (57.5)	808 (42.5)	1.1 (0.96–1.27)	0.16	1.27 (1.07–1.5)	0.007	781 (71.5)	311 (28.5)	0.62 (0.5–0.78)	< 0.001	0.82 (0.64–1.05)	0.11
Type of mask												
Surgical							770 (71.6)	305 (28.4)	Reference		Reference	
Cloth							652 (79.8)	165 (20.2)	1.56 (1.27–1.96)	< 0.001	1.53 (1.23–1.91)	< 0.001
Gender												
Female	1190 (61.2)	753 (38.8)	Reference		Reference		896 (75.3)	294 (24.7)	Reference			
Male	702 (49.8)	709 (50.2)	0.63 (0.55–0.72)	< 0.001	0.57 (0.49–0.65)	< 0.001	526 (74.9)	176 (25.1)	1.02 (0.82–1.27)	0.86		
Age category												
21–40 years	717 (48)	778 (52)	Reference		Reference		487 (67.9)	230 (32.1)	Reference		Reference	
41–65 years	724 (61.1)	460 (38.9)	1.71 (1.46–1.99)	< 0.001	1.7 (1.43–2.02)	< 0.001	578 (79.8)	146 (20.2)	1.87 (1.47–2.38)	< 0.001	1.75 (1.37–2.23)	< 0.001
> 65 years	451 (66.8)	224 (33.2)	2.18 (1.81–2.64)	< 0.001	2.28 (1.83–2.85)	< 0.001	357 (79.2)	94 (20.8)	1.79 (1.36–2.37)	< 0.001	1.52 (1.13–2.03)	0.005

Outcome was assessed using multivariate logistic regression estimating odd ratios (ORs) and 95% confidence intervals (CIs). Crude and adjusted odds ratios determine the factors associated with the use and the visual correct positioning of facemask fit. Values presented as number (%). *p* < 0.05 was considered statistically significant. The multivariate model had no missing data

Among the individuals wearing a facemask, correct positioning was significantly higher in rural (aOR, 1.38; 95% CI 1.07–1.79; *p* = 0.03), in indoor areas (aOR, 1.85; 95% CI 1.49–2.3; *p* < 0.001), in the 41–65 years age group (OR, 1.75; 95% CI 1.37–2.23; *p* < 0.001) and in the > 65 years age group (OR, 1.52; 95% CI 1.13–2.03; *p* = 0.005). The use of cloth masks in comparison with surgical masks was significantly associated with correct positioning (aOR, 1.53; 95% CI 1.23–1.91; *p* < 0.001). (Table 2).

## Discussion

In a post lockdown context with large clusters of COVID-19 cases leading to a potential second wave, only 56% of the individuals in the community wore a mask despite the recommendations and only three quarters of them wore it correctly. So less than half of the individuals were correctly protected in the general population.

Unsurprisingly, the mandatory process was the most powerful variable associated with increased use of facemasks. The mandatory approach may represent the best political lever to increase the level of facemask use in the general population. However, the mandatory wearing of facemasks did not significantly improve correct masking and therefore the infection control.

Among the people wearing a mask incorrectly, the most commonly observed positions were below the chin or below the nose. These observations suggest that facemasks are being handled and repositioned by individuals perhaps due to respiratory discomfort. These behaviors could lead to an increase in the risk of transmission, particularly through hand contamination. This fact is important due to the difficulty in complying with hand hygiene measures when putting the facemask on and taking it off. One hypothesis would be that mandatory universal facemasking, even in the absence of scientific evidence outdoors, would have the advantage of simplifying the measure and limiting mask handling and repositioning.

The positioning of cloth masks was significantly better in comparison with surgical facemasks. The characteristics of surgical facemasks (impersonal, single-use, more expensive, potentially less comfortable to wear) may decrease compliance with best practice. On the other hand, the good quality cloth masks with suitable sizes may fit better on the face making them more comfortable. The personalization of the designs of cloth facemasks could make them a fashion accessory allowing for better user compliance [3]. However, recent doubts were expressed in France regarding the capacities of “homemade cloth mask” to protect against SARS-CoV-2 contaminations [4].

The use of facemasks was significantly lower and more often worn incorrectly in the population < 40 years and in males independently of non-use of the mask. This finding is consistent with the increase in COVID-19 cases in the younger population during the post lockdown period [5, 6]. These populations represent a target for authorities in their information campaigns to optimize the protection of the general population.

Facemasks were worn correctly by those in rural areas compared with urban areas. In small cities, people are living together as part of an identifiable network, with significant social norms and better individual behaviors. In contrast, in urban populations, individuals are anonymous, with less reference to norms and altruistic measures. Further qualitative studies are needed to explore these assumptions.

To our knowledge, this study is the first to quantify the frequency and quality of the use of facemasks in the general population. However, this study has limitations: (i) the visual and potentially subjective evaluation of some criteria (correct masking, age category); (ii) the generalizability is questionable despite the inclusion of a range of situations at the regional scale; (iii) in the statistical analysis, due to the paucity of data in this context, we selected a cut-off for the multivariable analysis of 0.25; (iv) multiple observations at the same location could introduce a bias requiring the use of a mixed logistic regression model, even if they concern only 18% of the observations. Finally, observations were performed in public areas. However, indoors social interactions in the private sphere across individuals poorly complying with barrier precautions, including the use of facemask, represent a large risk of transmission.

## Conclusions

During the initial phase of the COVID-19 pandemic, the frequency and quality of facemask wearing remained low in the community setting. Young people in general, and men in particular, represent the priority targets for information campaigns. Simplifying the rules to require universal mandatory facemasking seemed to be the most effective approach for health authorities.

## Supplementary Information

**Additional file 1. Fig. S1**: Geographic location of the observation sites.

**Additional file 2. Fig. S2**: Definitions for the qualitative evaluation of mask position.

## Data Availability

Data sharing not applicable to this article because no datasets were generated or analyzed during the study.

## Abbreviations

OR: Unadjusted odds ratio
aOR: Adjusted odds ratio
