# Supplementary material for: How do the general population behave with facemasks to prevent COVID-19 in the community? A multi-site observational study
Source: Antimicrob Resist Infect Control. 2021 Mar 29;10:61. doi: 10.1186/s13756-021-00927-6 (PMC8006136; doi:10.1186/s13756-021-00927-6)
Supplement: Supplementary file 1 — Additional file 1. Fig. S1: Geographic location of the observation sites. [file 13756_2021_927_MOESM1_ESM.docx]

Additional Figure 1. Geographic Location of the Observation Sites

**
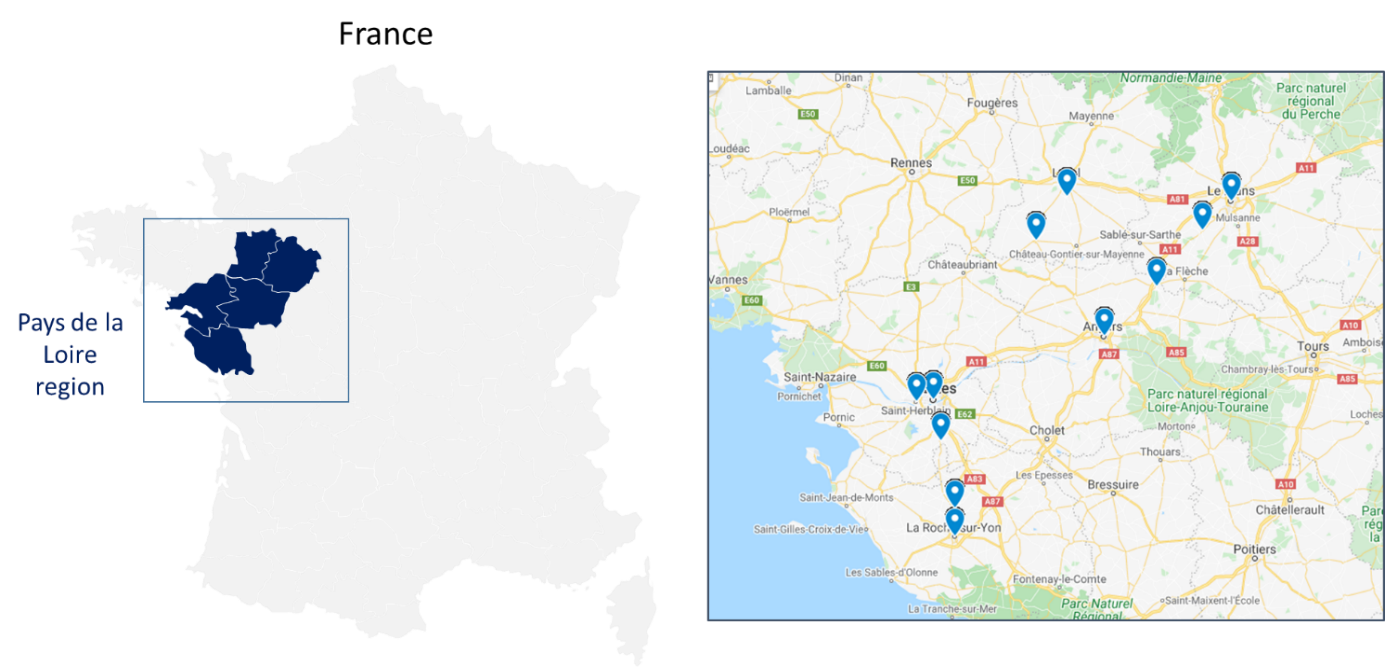
**

**List of cities :** Allones, Angers, Belleville sur Vie, Craon, Durtal, Geneston, Laval, Le Mans, L'huisserie, Nantes, La Roche sur Yon, Saint Herblain, Suze sur Sarthe
