# Supplementary material for: How do the general population behave with facemasks to prevent COVID-19 in the community? A multi-site observational study
Source: Antimicrob Resist Infect Control. 2021 Mar 29;10:61. doi: 10.1186/s13756-021-00927-6 (PMC8006136; doi:10.1186/s13756-021-00927-6)
Supplement: Supplementary file 2 — Additional file 2. Fig. S2: Definitions for the qualitative evaluation of mask position. [file 13756_2021_927_MOESM2_ESM.docx]

Additional Figure 2. Definitions for the Qualitative Evaluation of Mask Position

| **Correct positioning** |  |
| --- | --- |
| Correct: bar fitted on the nose, mask stretched to the chin, the fasteners are all used and are not crossed | 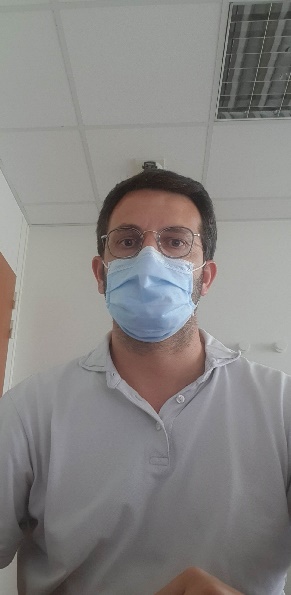 |
| **Incorrect positioning** |  |
| Below the nose: the mask only covers the mouth, the nose is visible | 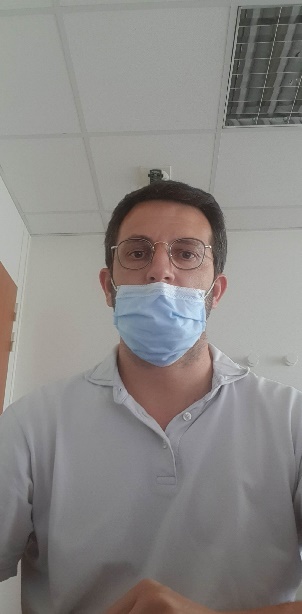 |
| Below the mouth: the mask is lowered, placed under the nose and mouth at the chin or neck | 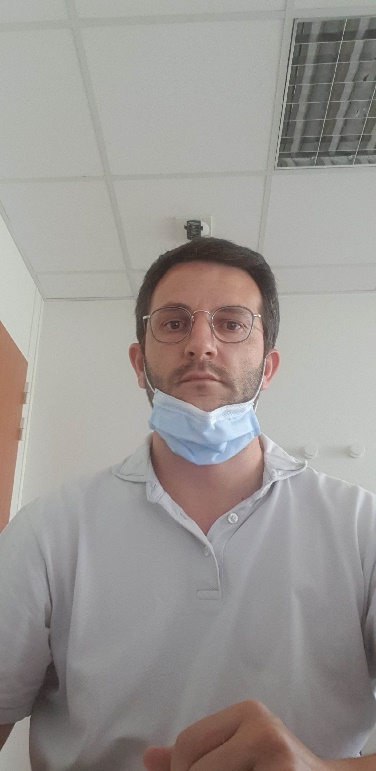 |
| On the forehead: the mask is raised and placed over the eyes | 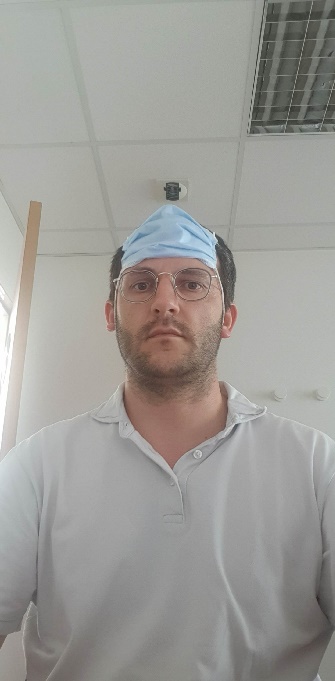 |
| On one ear: the mask is worn only by a rubber band hanging from one ear | 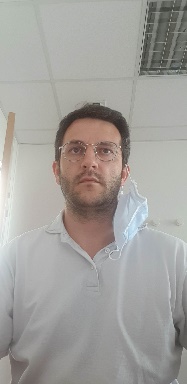 |
| Worn backward (outside in): the colored side of the mask is inside rather than outside | 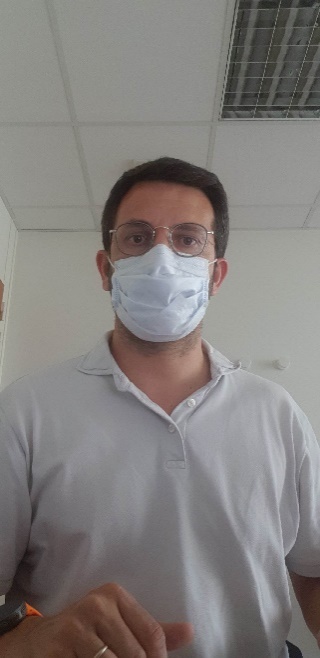 |
| No adjustment of the bar on the nose: the mask is placed on the nose but is not fitted around it | 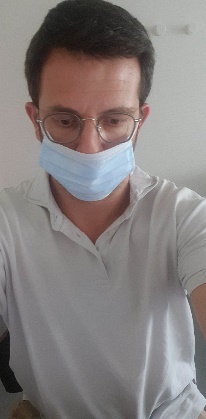 |
| Not stretched under the chin: the mask does not cover the chin | 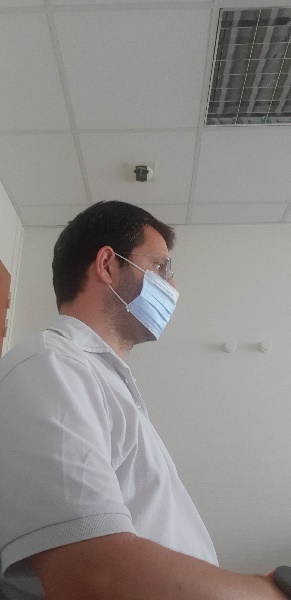 |
| Cross fasteners (twisted elastic, strap from top to bottom): the elastic bands or straps cross | 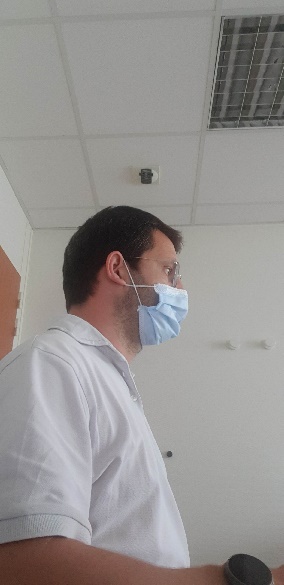 |
| Partial attachment with only one strap on each side: only the top straps are tied | 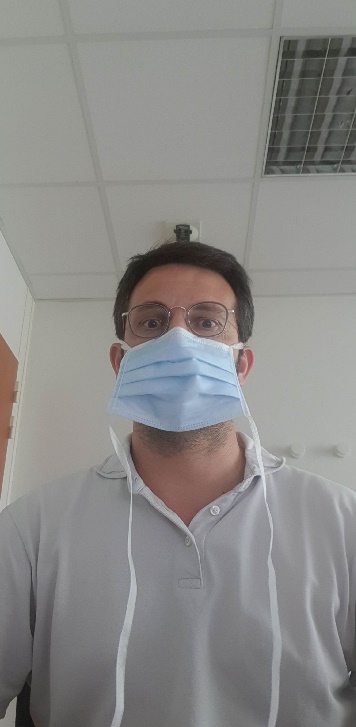 |
| Long hair falling on the mask or face (not tied): long hair covers part of the mask | 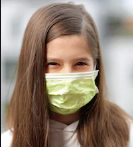 |
